# Improving experimental phases for strong reflections prior to density modification

**DOI:** 10.1107/S0907444913018167

**Published:** 2013-09-20

**Authors:** Monarin Uervirojnangkoorn, Rolf Hilgenfeld, Thomas C. Terwilliger, Randy J. Read

**Affiliations:** a Institute of Biochemistry, Centre for Structural and Cell Biology in Medicine, University of Lübeck, Ratzeburger Allee 160, 23538 Lübeck, Germany; b Graduate School for Computing in Medicine and Life Sciences, University of Lübeck, Ratzeburger Allee 160, 23538 Lübeck, Germany; c Shanghai Institute of Materia Medica, Chinese Academy of Sciences, 555 Zu Chong Zhi Road, Shanghai 201203, People’s Republic of China; d Bioscience Division and Los Alamos Institutes, Los Alamos National Laboratory, Los Alamos, NM 87545, USA; e Department of Haematology, Cambridge Institute for Medical Research, University of Cambridge, Cambridge CB2 0XY, England

**Keywords:** experimental phasing, density modification, genetic algorithms

## Abstract

Experimental phasing of diffraction data from macromolecular crystals involves deriving phase probability distributions. These distributions are often bimodal, making their weighted average, the centroid phase, improbable, so that electron-density maps computed using centroid phases are often non-­interpretable. Density modification brings in information about the characteristics of electron density in protein crystals. In successful cases, this allows a choice between the modes in the phase probability distributions, and the maps can cross the borderline between non-interpretable and interpretable. Based on the suggestions by Vekhter [Vekhter (2005 ▶), *Acta Cryst.* D**61**, 899–902], the impact of identifying optimized phases for a small number of strong reflections prior to the density-modification process was investigated while using the centroid phase as a starting point for the remaining reflections. A genetic algorithm was developed that optimizes the quality of such phases using the skewness of the density map as a target function. Phases optimized in this way are then used in density modification. In most of the tests, the resulting maps were of higher quality than maps generated from the original centroid phases. In one of the test cases, the new method sufficiently improved a marginal set of experimental SAD phases to enable successful map interpretation. A computer program, *SISA*, has been developed to apply this method for phase improvement in macromolecular crystallo­graphy.

## Abstract 2

A genetic algorithm has been developed to optimize the phases of the strongest reflections in SIR/SAD data. This is shown to facilitate density modification and model building in several test cases.

## 1. Introduction

Experimental SAD phasing allows us to obtain phase information by solving equations based on differences between Friedel pairs of structure factors. The possible solutions for a reflection are represented in the form of a probability distribution (Blow & Crick, 1959 ▶; Otwinowski, 1991 ▶; McCoy *et al.*, 2004 ▶). Towards solving a structure, this phasing information is passed on to density modification, which exploits the expected features of molecular maps to break the ambiguity that exists in the initial distribution (Wang, 1985 ▶). In the case where many reflections have accurate phases, obtaining an interpretable map is straightforward. In contrast, when the majority of the reflections are poorly determined, resolving the ambiguity remains a difficult task.

We selected a SAD data set from gene V protein (Skinner *et al.*, 1994 ▶) as an example of this situation. Solving this structure from just the peak-wavelength SAD data is challenging owing to the low quality of the electron-density map obtained after density modification. However, the structure could be solved from a MAD data set. This is a common situation when experimental phases result in a poor map.

Vekhter (2005 ▶) presented an interesting study in which it was shown that by assigning low-error phases to a few of the strongest reflections, the entire set of phases could become significantly improved after density modification. There were five structures with 5000–17 000 reflections in the test and it was very encouraging to see that such large data sets could be improved by having only the 124 strongest reflections assigned the correct phase. Vekhter (2005 ▶) assigned correct phases calculated from the model and proposed that in practice phases could be measured experimentally by a three-beam diffraction experiment. Here, we follow up this analysis by exploring computational methods to select improved phases for a few of the strongest reflections before feeding them into density modification. We addressed the following points to pursue this goal.

(i) We tested whether the map skewness (Podjarny & Yonath, 1977 ▶), which describes the extent to which the extreme values in a map tend to be systematically positive or negative, could be used to identify the correct phases for a few of the strongest reflections. We performed this test by developing a genetic algorithm that searches for combinations of phases for the strongest reflections. In the presence of the entire data set, these led to better values of the skewness. We observed that correct phases for the strongest reflections correlate with increasing values of the map skewness. A computer program, *SISA* (‘SIR/SAD phase optimization’), has been written that will be incorporated into the *PHENIX* software package (Adams *et al.*, 2010 ▶).
(ii) For three ‘difficult structures’, we tested the efficiency of having the skewness as a target function to implement the algorithm that optimizes the phase quality for the strongest reflections. In order to observe the effect of this improvement, we used the optimized data set for density modification and compared the results with those obtained from density modification using the original data.

We selected the data sets presented in Table 1 ▶ because they were borderline cases in which density-modified phases were not good enough to generate an interpretable map.

**Figure 1:**
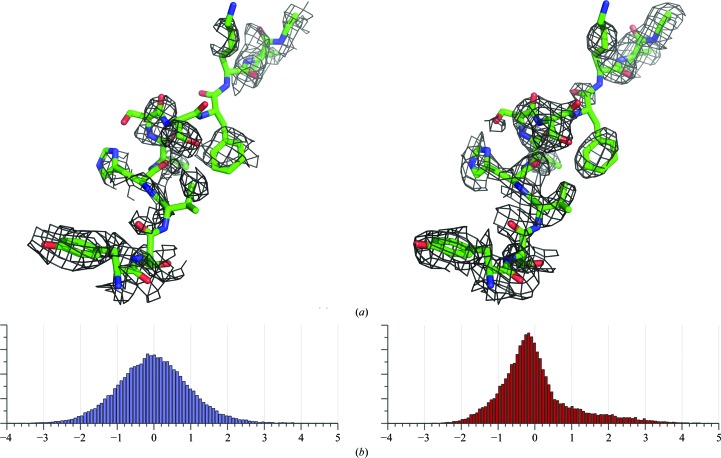
Two key ideas exploited in the implementation of the method. (*a*) A comparison of two density-modified maps generated from SAD data for the gene V protein: the first map was derived from a reflection set with the original centroid phases (ϕ_B_), while the second map was derived by assigning correct phases (from the PDB model) to the 100 strongest reflections from the same data set. The map correlation of the second map was significantly improved from 0.45 to 0.66. (*b*) A comparison of two electron-density histograms: the histogram on the left was generated from the electron-density map calculated using the centroid phases (ϕ_B_) resulting in a small value of map skewness (skew = 0.22; see equation 1), while the histogram on the right was generated from the map calculated using the correct phases (ϕ_C_), resulting in a large value of map skewness (skew = 1.11).

There are two key ideas that we exploit here in order to improve the quality of the experimental maps. The first involves the role of the strongest reflections. We tried replacing the centroid phases of the 100 strongest reflections with correct phases (calculated from the PDB model) for one of our test cases, the gene V protein; we passed this set of reflections on to density modification and calculated a map correlation as defined in equation (2) (see below; Read, 1986 ▶; Lunin & Woolfson, 1993 ▶) for the density-modified map. In line with the observations of Vekhter (2005 ▶), the map correlation of the new set of structure factors, consisting of the 100 strongest reflections with optimized phases and the remaining reflections with unmodified phases, increased from 0.45 (the value obtained when the original reflection set was used) to 0.66 (Fig. 1 ▶ *a*).

The importance of these strongest reflections can also be appreciated by noting that the mean-square error in electron density introduced by a phase error is proportional to the squared amplitude of the reflection. The 100 strongest reflections (only 4% of all reflections) of gene V protein contribute 23% of the sum of squared amplitudes for the whole data set. This also indicates that there is a limited number of the strongest reflections that can be improved, because there will be diminishing returns in the sum of squared amplitudes if more reflections are included. As is to be expected, this 23% contribution to the sum of squared amplitudes is not distributed uniformly among different resolution shells; 64% of this 23% share of the total sum of squared amplitudes is contributed by reflections in the lowest resolution shell (>10 Å).

Considering that a few of the strongest reflections can have an impact on density modification, it is possible to implement algorithms that search for phase combinations in this compact solution space. As we have knowledge about phases from experimental phasing, there is no need to search the entire range of values from 0 to 2π, but we can limit the choice of phases for a reflection based on its probability distribution.

The second key idea is based on measures of molecular map quality. Note that we are going to choose alternative phases for only a few of the strongest reflections. The rest of the reflections will be used with their original centroid phase and any new map will be calculated using the complete set of reflections. In this way, the phases for the reflections that are not varied provide a background of known information used for the map calculation, and the phases that are varied are being tested for consistency with the other phases. The newly generated maps are assumed to have some molecular features as a starting point that we could use to calculate a measure of map quality. We chose the skewness of the density values in an electron-density map in this work, as it was pointed out by Terwilliger *et al.* (2009 ▶) that it was the most accurate indicator for estimating map quality out of ten measures tested. We set the skew function (1) as our target function for the search algorithm,
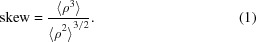
Fig. 1 ▶(*b*) shows a comparison of the electron-density histogram generated from phases from the SAD data (*ϕ*_B_) and phases from the solved structure (ϕ_C_) for the gene V protein. Electron-density maps for the two sources of phase were generated accordingly and a threshold of ±5σ was applied for the density cutoff in the maps. The skewness was calculated using (1) and values of about 0.22 and 1.11 were obtained for the first and the second case, respectively. It is necessary to apply the threshold cutoff to truncate the density map, since most of the starting experimental maps tend to have some highly positive and negative values. The truncation helps to prevent extreme map-skewness values resulting from a few very large peaks.

## 2. The method

We chose a genetic algorithm as the optimizing tool because of the useful features of such algorithms in problem representation and search-space exploration. Genetic algorithms were pioneered by Holland (1975 ▶) and have been implemented as search tools in a variety of methods in X-ray diffraction such as small-angle scattering to determine the shapes of molecules (Franke & Svergun, 2009 ▶), powder diffraction to recover phases (Shankland *et al.*, 1997 ▶; Harris *et al.*, 2004 ▶; Feng & Dong, 2007 ▶) and *ab initio* phasing in macromolecular crystallography at low resolution (Miller *et al.*, 1996 ▶; Webster & Hilgenfeld, 2001 ▶; Zhou & Su, 2004 ▶; Immirzi *et al.*, 2009 ▶).

**Figure 2:**
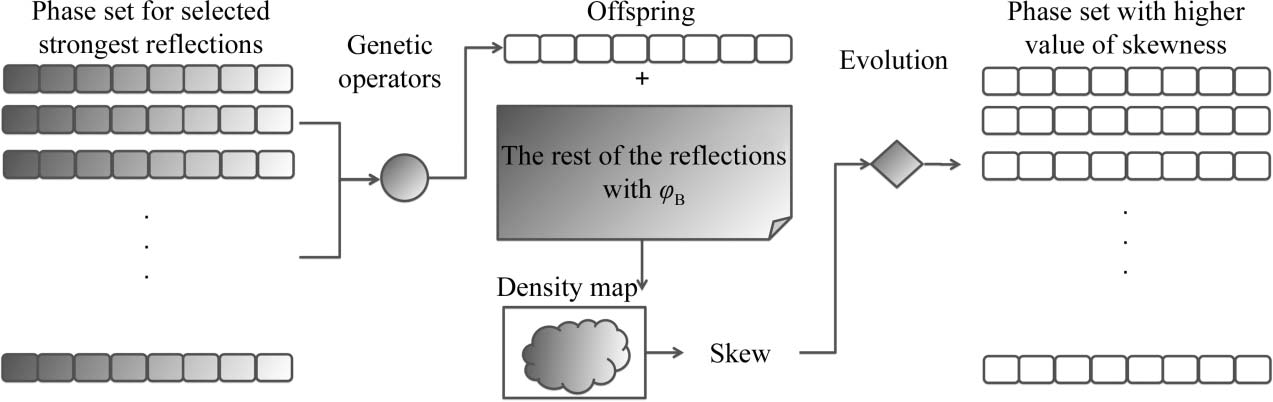
Implementation of the genetic algorithm.

Our implementation takes the phase probability distributions of the strongest reflections selected as input, creates a data structure analogous to chromosomes to store these phases, manipulates each chromosome by genetic operators, selects only those with a higher skew value than the parents and outputs the solutions with a high value of the target function (Fig. 2 ▶). At the end of each run, we measure two quantities: (i) the map correlation (equation 2; see below) between the optimized phases (ϕ_S_) and the calculated phases from the correct model (ϕ_C_) for only the strongest reflections selected in the search and (ii) the map correlation between the density-modified map (generated by combining the optimized phases for the selected reflections with the centroid phases for the rest of the reflections and passing them on to density modification; all reflections are used in the process) and the calculated map (generated from the solved structure).
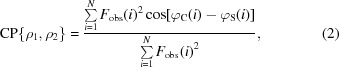
where *N* is the number of selected reflections.

We divided our implementation of the *SISA* procedure into three parts: firstly, initializing the phase choices (stored in a chromosome) from the phase probability distribution function; secondly, applying a genetic algorithm and genetic operators, with the target function being the skewness of the density map; and thirdly, selecting the best solution, assigning new figures of merit and passing them on to density modification and model building. All parts of the algorithms were written in Python together with the usage of the *cctbx* libraries (Grosse-Kunstleve *et al.*, 2002 ▶).

### 2.1. Initialization of the phase choices

**Figure 3:**
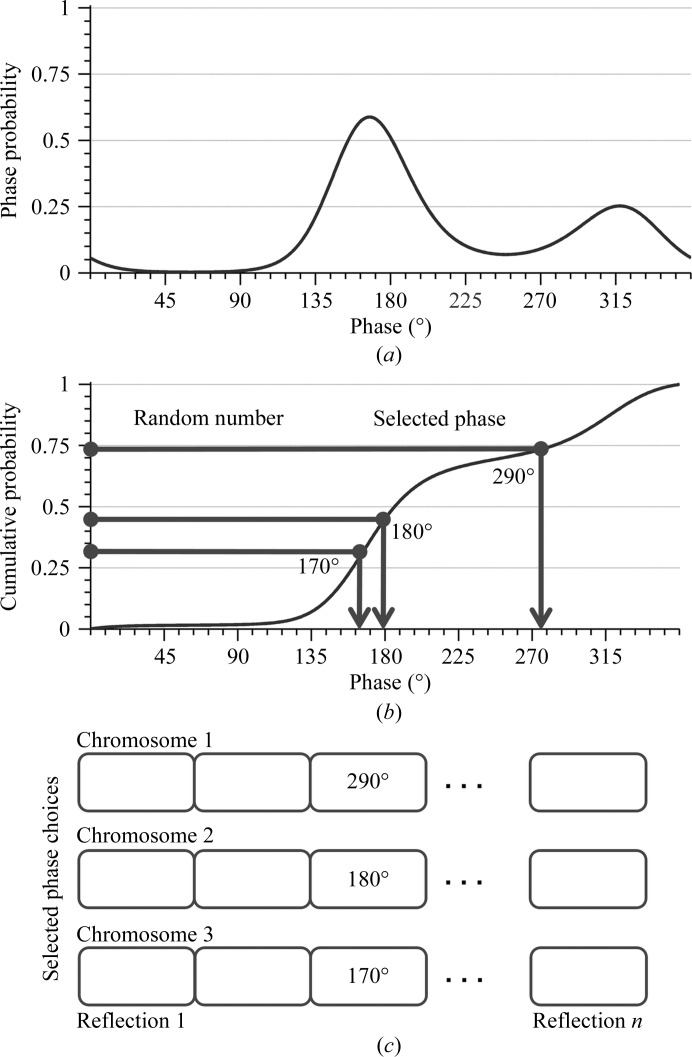
Selection of phase choices other than ϕ_B_ for a reflection. (*a*) Phase-probability distribution function. (*b*) Cumulative distribution function calculated from (*a*). (*c*) Chromosomes storing phase choices for the genetic algorithm.

We generated phase choices for a reflection according to its phase probability distribution function encoded in the Hendrickson–Lattman coefficients (Hendrickson & Lattman, 1970 ▶). An example of selecting a phase for a reflection is shown in Fig. 3 ▶. In the case of this bimodal distribution, a centroid phase (ϕ_B_) would traditionally be selected. In our method, we allowed phases from the phase probability distribution to be selected. In practice, we converted the phase probability distribution (Fig. 3 ▶ *a*) to a cumulative distribution (Fig. 3 ▶ *b*). The algorithm is equivalent to picking a random number in the range 0–1, drawing a line horizontally to intersect with the cumulative function and selecting the phase at this point of the curve. By doing this many times, we could sample all possible choices of phase for that reflection. It is also clear that those phases with higher probability are most likely to be selected because of the large slope of the cumulative function. At the end, we generated a number of phase choices according to our desired number of density maps (the size of the population for the genetic algorithm). This process was carried out for each of the selected reflections.

Note that we only applied these alternative choices to varying numbers of the strongest reflections. The rest of the reflections, which comprised the majority, maintained the centroid phases (ϕ_B_). Even though the phases of the remaining reflections were not perturbed, they play an important role in interacting with the varied reflections to determine the skew value. We show in §[Sec sec3]3 that phase improvements could only be obtained when the varied reflections are used with the other reflections to generate the density map and calculate the map skewness as the target function for the search.

### 2.2. The genetic algorithm

The second part is the implementation of the genetic algorithm. This type of stochastic search algorithm has two important features. The first feature is the way that the information representing the possible solution to the problem is stored. The genetic algorithm treats each set of answers as a chromosome, which looks like the output that we have just constructed from the first part (Fig. 3 ▶ *c*), where each phase value is a possible answer for a reflection. Note that the values stored in the chromosomes are not represented by binary strings but by the set of non-negative integers from 0 to 359. Our algorithm treats these many combinations of phases that we have just created as a starting pool of chromosomes.

The second feature comprises the selection and recombination process. In order to increase search performance, we chose the geographical-restraint technique (Connor, 1994 ▶) over the probability-weighted (also known as roulette-wheel) method (Bäck *et al.*, 1997 ▶) for this selection process. This decision was based on a comparison of the search performance by using the SAD data from gene V and employing these two selection techniques to search for phases for the 100 strongest reflections. The genetic algorithm was set to terminate when all chromosomes in the population pool became homogeneous (the average of the map correlations calculated from all pairs of chromosomes in the same generation was >0.9). We needed around 95–97 generations when using the roulette-wheel selection but only around 9–11 generations with the geo­graphical restraint for the termination of the algorithm. Both selection techniques resulted in a similar quality of the map correlation (around 0.53) calculated from the 100 optimized phases.

**Figure 4:**
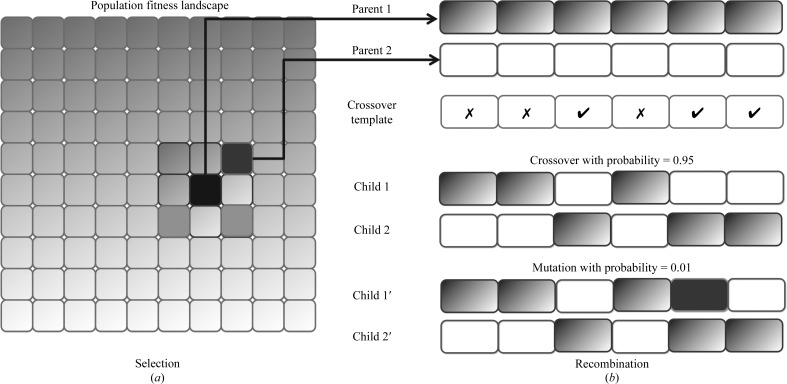
Geographical-restraint technique used in the selection and recombination process for the genetic algorithm. (*a*) A parent is selected (black location) from a random location on the fitness landscape, where a local map is drawn around it. By performing random walks on this local map, more chromosomes are selected as candidates (medium grey) and the fittest one (dark grey) is chosen for the recombination process. (*b*) A pair of selected chromosomes is chosen for recombination under the control of probability of crossover and mutation. The uniform crossover technique was used for the crossover operation, where only locations indicated on the crossover template were exchanged between the parents. The mutation operator occurred on randomly selected locations on the child chromosomes where their phases were replaced by new phases redrawn from the phase probability distribution.

Fig. 4 ▶ illustrates how the geographical-restraint technique was implemented for the selection process. At any time, a parent chromosome is selected from a random location on a map where another smaller map is drawn to cover the selected position (Fig. 4 ▶ *a*). The algorithm performs random walks on this smaller map to select candidates for recombination and chooses the one with the highest fitness value. In comparison with the roulette wheel, where only a group of chromosomes with high fitness values is likely to be selected at any given time, the geographical-restraint method allows chromosomes at different locations on the fitness landscape to be selected for the recombination process even though they do not belong to the group with high fitness values. This prevents a premature loss of diversity in the population.

The evolution process is carried out by the application of a mutation operator and recombination of the parents with a crossover operator. These two mechanisms are controlled by the probabilities of crossover and mutation accordingly, so that many of the fitter solutions and some unfit solutions would be selected for the next generation.

For the recombination process, we chose uniform crossover, which allows randomly selected segments from the parents’ chromosome to be exchanged (Sywerda, 1989 ▶). It was suggested as a suitable operator for problems with complex search spaces in which the practical population size could not meet the necessary sampling accuracy (De Jong & Spears, 1991 ▶), which might be the case for this work. In our problem setting, one way to imagine the size of the solution space is to consider the number of phase sets that must be tested for 1000 reflections. If each reflection has two choices for the phase (as in the case of the bimodal distribution), there are 2^1000^ combinations of phases to be tested in order to obtain the correct answer. In order to still be able to compute some answer, our approach only generates around 400 combinations of phases per test run and this number is much smaller than the number required to obtain a correct answer: the uniform crossover encourages disruption of the chromosomes, which may help the algorithm to explore more possibilities for optimized phases.

An example of how the recombination process works with our method is illustrated in Fig. 4 ▶(*b*). From the population pool, a pair of phase sets is selected. In order to recombine their chromosomes, a random template is generated indicating locations where the genes will be swapped. This template is newly created every time crossover occurs. With a certain probability, some of the genes of these two new offspring chromosomes are also mutated. When mutation occurs, the algorithm randomly selects a new phase from the original phase probability distribution of the reflection. The target function is then recalculated from the new phase combination. The parent pair is replaced by its offspring only when the latter has a higher value for the target function.

### 2.3. The composite solution

The last part of the process concerns the selection of the best solution from the optimization process. To sample solution space, several independent microruns were carried out so that many solutions from different starting points could be obtained. Once all runs were completed, we noticed that there were different solutions that could produce similarly high values of map skewness, *i.e.* for a selected value of map skewness the value of phase difference between the best solution and the worst solution could be up to around 15°. In order to avoid selecting the worst solution, those solutions for which the value of the fitness was higher than the average value were selected and their centroid phases were calculated as the best solution. This composite best solution is the output from each run of the search process.

We combined these optimized phases and updated figures of merit with the centroid phases and their original figures of merit and then measured the impact of optimizing the strongest reflections by feeding this new set of reflections to density modification. As discussed below (see Fig. 6), figures of merit for the selected reflections were either updated by increasing the initial value by 0.2 or left unchanged.

Throughout each run, the genetic algorithm was controlled by the following parameters.

(i) *N*_chromosomes_, the number of chromosomes.
(ii) *N*_generations_, the number of generations.
(iii) *P*_cross_, the probability of crossover (0.0–1.0).
(iv) *P*_mutate_, the probability of mutation (0.0–1.0).
(v) *R*_crosspoints_, the number of crossover points represented by a fraction of the chromosome size.
(vi) *N*_mutatepoints_, the number of mutation points.

These parameters determine the size of the solution space that each run can represent and the amount of computing time required.

## 3. Results and discussion

### 3.1. Case I: gene V protein

The SAD data set from this crystal yielded phases with a mean figure of merit of 0.42 for the entire set of reflections. By supplying the data set with the sequence of the molecule to an automatic model-building program, *PHENIX AutoBuild*, we could obtain a model at the end of the run with 42 out of 87 residues built with *R* = 0.46 and a map correlation of 0.52. The data set (about 2500 reflections) was collected from a crystal belonging to space group *C*2 with unit-cell parameters as shown in Table 1 ▶.

There are two points that directed our test procedures here. We were interested to determine whether the skew function could be used to improve the phases of a few strongest reflections and, if so, to determine whether the new phases could make an impact on the density modification. To meet the first goal, we chose to run the optimization algorithm for varying numbers of strongest reflections. Apart from the different numbers of reflections, we assigned the same parameters (*N*_chromosomes_ = 400, *N*_generations_ = 100, *P*_cross_ = 0.95, *P*_mutate_ = 0.01, *R*_crosspoints_ = 0.2 and *N*_mutatepoints_ = 1) to the genetic algorithm for all of the runs and terminated the procedure when every pair of phase sets in the chromosome had a map correlation of >0.9. This set of values for the parameters was selected among various sets of test values because it appeared to yield optimum results while retaining satisfactory computing performance. To observe the changes in phase quality, we calculated the map correlations (2) for a particular chromosome, which stored phase choices for the selected reflections, in comparison to the known ϕ_C_.

**Figure 5:**
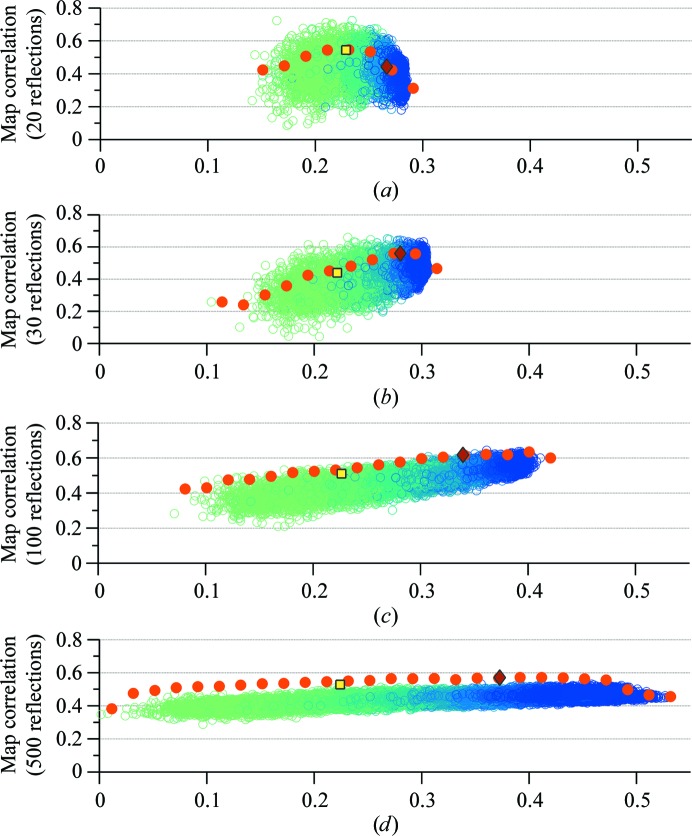
Measures of the quality of the optimized phases for the strongest reflections selected in the search. The measures were calculated using the map correlation coefficients (equation 2) of the optimized phases (ϕ_S_) and the known phases (ϕ_C_). Each dot represents a set of phases yielding a certain skew value of the density map and a certain value of map correlation. The filled dots show the map correlation coefficients of the centroid phases calculated from a group of phases with similar skew value. All plots show the results from five independent runs, with a square marker representing the original centroid phases ϕ_B_ and a diamond marker representing the optimized phases ϕ_S_ selected as the output of the search process for (*a*) the 20 strongest reflections, (*b*) the 30 strongest reflections, (*c*) the 100 strongest reflections and (*d*) the 500 strongest reflections.

We tested the optimization procedure by selecting the 20, 30, 100 and 500 strongest reflections. To measure the quality of the phases, we generated a scatter plot (Fig. 5 ▶) between the map correlation calculated using only the selected reflections in the search (vertical axis) and the map skewness (horizontal axis) with one particular point representing a set of phases for selected reflections. The colour, which changes from light green to dark blue, represents the number of generations of optimization that were necessary. The square and diamond markers represent ϕ_B_ and the optimized phases ϕ_S_, respectively. Note that ϕ_S_ is the new centroid phase calculated from the selected chromosomes that have a skew value greater than the average. These plots also reveal the variation of overall phase quality during the optimization process, as can be seen from the series of filled dots. Each filled dot represents the phase quality of the centroid phases computed from a collection of phase sets with similar skew values. These centroid phases tend to have higher phase quality than the individual samples, as evident in particular when larger numbers of reflections are varied.

**Figure 6:**
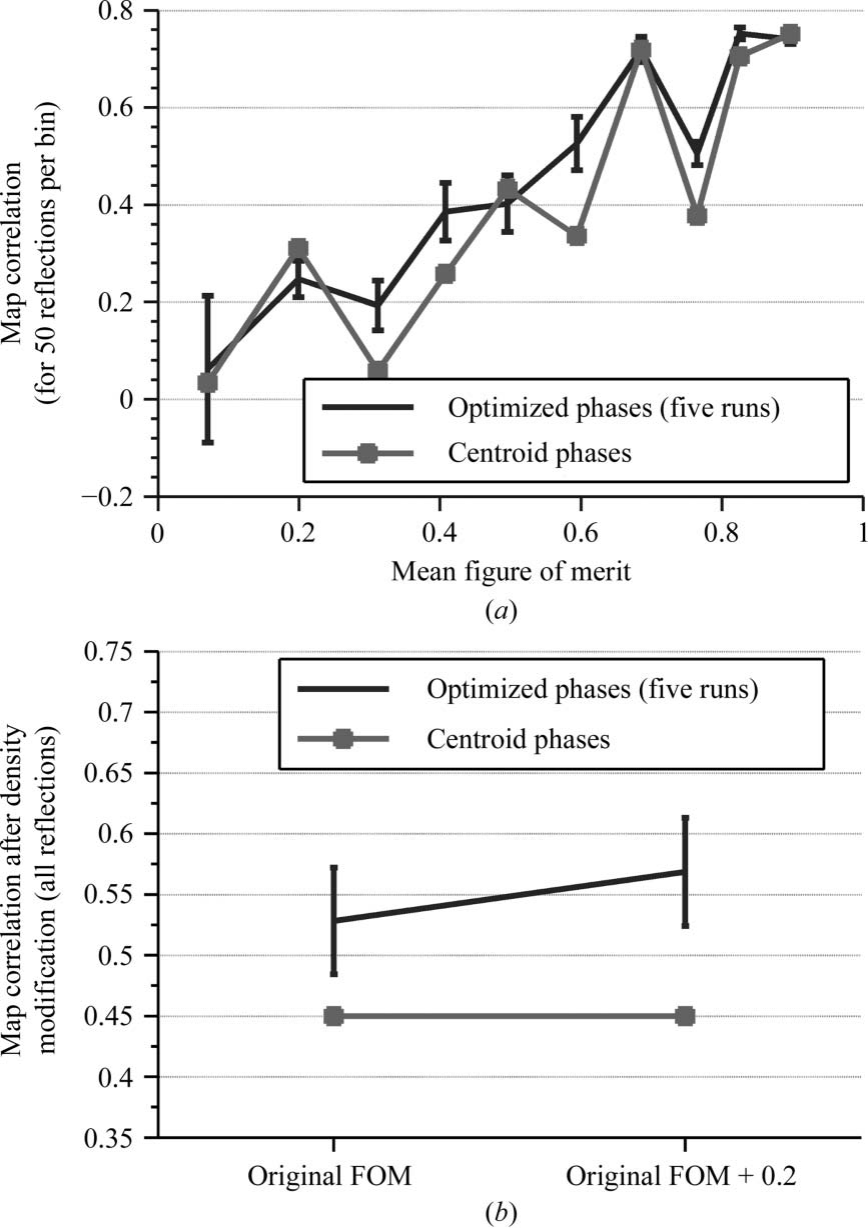
(*a*) Quality of the optimized phases for the 500 strongest reflections grouped according to their original figure of merit. (*b*) A comparison of the quality of density-modified maps generated using the original figures of merit and the original figures of merit increased by 0.2.

These plots tell us that at least 30 strongest reflections should be selected in order to obtain phase improvements, because with this minimum number of reflections chosen we were able to obtain optimized phases (ϕ_S_) with better map correlations than those calculated using the centroid phases (ϕ_B_). As we increased the number of selected reflections, we observed that the algorithm achieved higher values of map skewness with less overall average improvement in phase quality for the varied reflections. In addition, we also noticed that most of this improvement occurred for reflections with a figure of merit larger than 0.2 (Fig. 6 ▶ *a*). We omitted these reflections from our subsequent tests.

The next step is to test the impact of these optimized phases on density modification. Here, we tried two ways to use the figure of merit for the strongest reflections selected: the original figures of merit and a slight increase of the original figures of merit (+0.2). It was possible to obtain improvements after density modification with the original figures of merit, but a slight inflation of the figures of merit led to even better results (Fig. 6 ▶ *b*). Another possibility is to compute the figure of merit from the distribution of phase values among the chromosomes (which store phases for the genetic algorithm) selected at the end of the search process; however, the genetic algorithm converged with a population with very similar phase values for each reflection, giving a distribution that peaked at the optimized phase (figure of merit close to 1).

**Figure 7:**
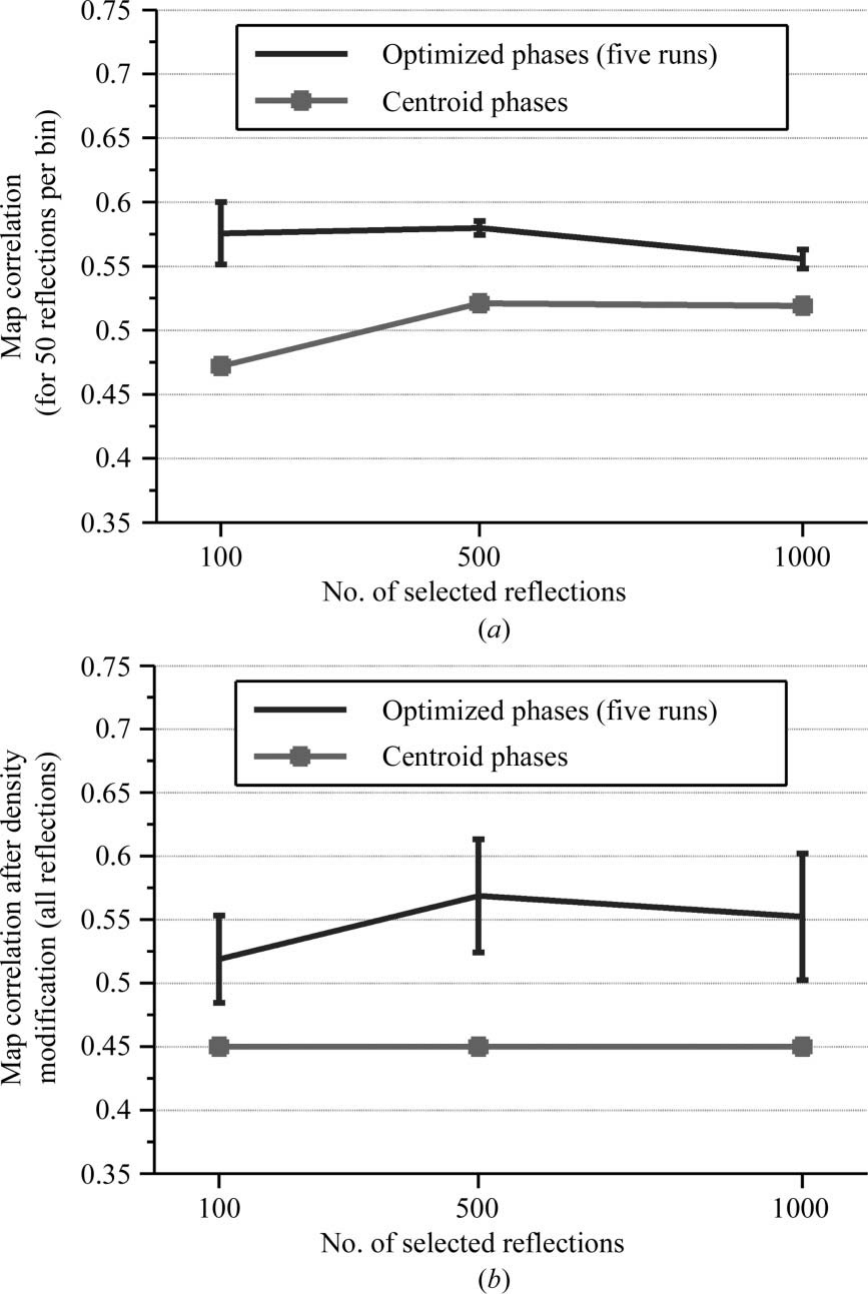
Results of running five independent trials on SAD data from the gene V protein to search for the phases of 100, 500 and 1000 selected strongest reflections. (*a*) Map correlations of only the selected reflections for the optimized phases. (*b*) Map correlations of all reflections for the density-modified maps.

Iterative searching helped to improve the quality of phases when optimizing the phases of more than 100 reflections; for 500 reflections, searching for phases for 100 reflections incrementally resulted in an averaged map correlation of 0.56 (for 500 selected reflections) from five independent runs (the map correlation of ϕ_B_ was 0.48 and the averaged map correlation of the optimized phases without iteration was 0.51; see Fig. 7 ▶ *a*).

Using the iterative search mode, we performed five independent runs for the 100, 500 and 1000 strongest reflections. We measured the map correlations of the optimized phases (ϕ_S_) for the selected reflections (Fig. 7 ▶ *a*) and combined these selected reflections (with the optimized phases) with the rest of the reflections (with the original centroid phases) for density modification. We calculated the map correlations of the density-modified phases for all reflections to observe the impact of the selected strongest reflections and their optimized phases (Fig. 7 ▶ *b*).

The results in Fig. 7 ▶ are grouped according to the number of strongest reflections; the error bar shows the mean and ±1σ of the map correlations obtained from five independent runs. For each group, the quality of ϕ_B_ for the selected number of reflections is shown using a square marker.

The optimized phases for all of the tests (with 100, 500 and 1000 strongest reflections) improved the quality of the density-modified maps; the map correlation increased from 0.45 (density-modified map using ϕ_B_) to averaged map correlations (from five runs) of 0.52, 0.57 and 0.55, respectively. Iteratively improving the quality of the phases was still possible even when the 1000 strongest reflections were selected; however, the density-modified phases (all reflections) for the 500 strongest reflection cases were sufficient to gain an improvement in the subsequent model-building cycles. We performed around 20 cycles of automatic model building using *PHENIX AutoBuild* for the density-modified map with the lowest and highest map correlation and obtained final maps with map correlations of 0.75 and 0.8 (the original centroid phases resulted in a map correlation of 0.52); the best map delivered an almost complete structure (84 out of 87 residues were found with an *R* and *R*_free_ of 0.20 and 0.27, respectively).

**Figure 8:**
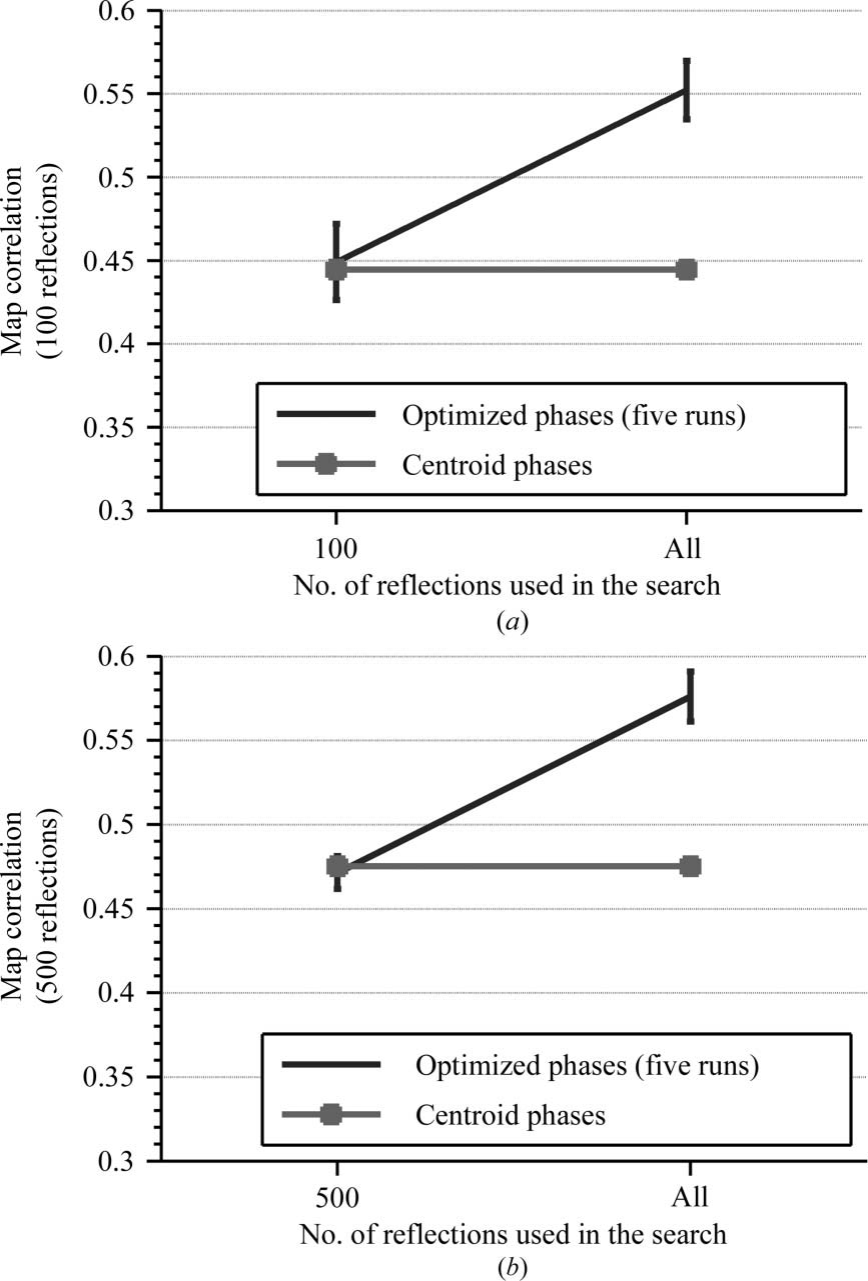
Comparisons of map correlations of the optimized phases (ϕ_S_) from the tests using the varied and the remaining reflections with map correlations from the tests using only the varied reflections for the calculation of map skewness as the target function for the search. The error bar on the plot indicates average and ±1σ map correlations obtained from five runs. The square marker indicates map correlation calculated using ϕ_B._ (*a*) The 100 strongest reflections case. (*b*) The 500 strongest reflections case.

The remaining reflections that were kept unvaried play an important role in obtaining phase improvement for the varied reflections. We ran two tests for the 100 and 500 strongest reflections: one using only the selected strongest reflections and another using all reflections to calculate the map skewness during the search (but with only the selected strongest reflection varied). Results for both the 100 and the 500 strongest reflections show that phase improvement could only be obtained when we used all of the reflections to generate the density map and calculate map skewness (the target function of the search); the map correlation increased to an average value of 0.55 (for five runs) for the 100 strongest reflections (Fig. 8 ▶ *a*) and to 0.57 for the 500 strongest reflections (Fig. 8 ▶ *b*); no improvement was observed when only the selected strongest reflections were used in the search.

We tried to investigate whether increasing the population size in the genetic algorithm could help to improve the results when optimizing the phases of more than 100 reflections. We performed ten runs with an increase in the population size from 400 to 2500 to search for the phases of the 500 strongest reflections. Leaving other parameters for the search at the same values as used previously, we obtained similar values of map correlation for the 500 strongest reflections as when a population size of 400 was used in the tests.

### 3.2. Cases II and III

The improvement after density modification and model building for the SAD data set of the gene V protein shows that map skewness can be used as a target function to search for more accurate phases than ϕ_B_. In order to investigate whether the same method can be applied to other cases, we selected two further data sets (cases II and III in Table 1 ▶) which had failed to give complete structures after density modification and model building.

The same protocol was applied to these two data sets as for the gene V protein. We first searched for phases for the 100, 500 and 1000 strongest reflections (using an iterative search for the latter two) using the genetic algorithm and obtained results from five independent runs in each test. We calculated the map correlation coefficients for all of the optimized phases (ϕ_S_) in comparison to the known structure (ϕ_C_), which was solved using different data sets. After the search operations were complete, we recombined the new set of phases for the selected reflections (inflating their original figures of merit slightly by adding 0.2) with the original centroid phases (ϕ_B_) of the remaining reflections and passed them on to density modification (using *PHENIX AutoBuild*). We collected the map correlations of the density-modified maps from the runs to investigate the impact of the new phase sets.

**Figure 9:**
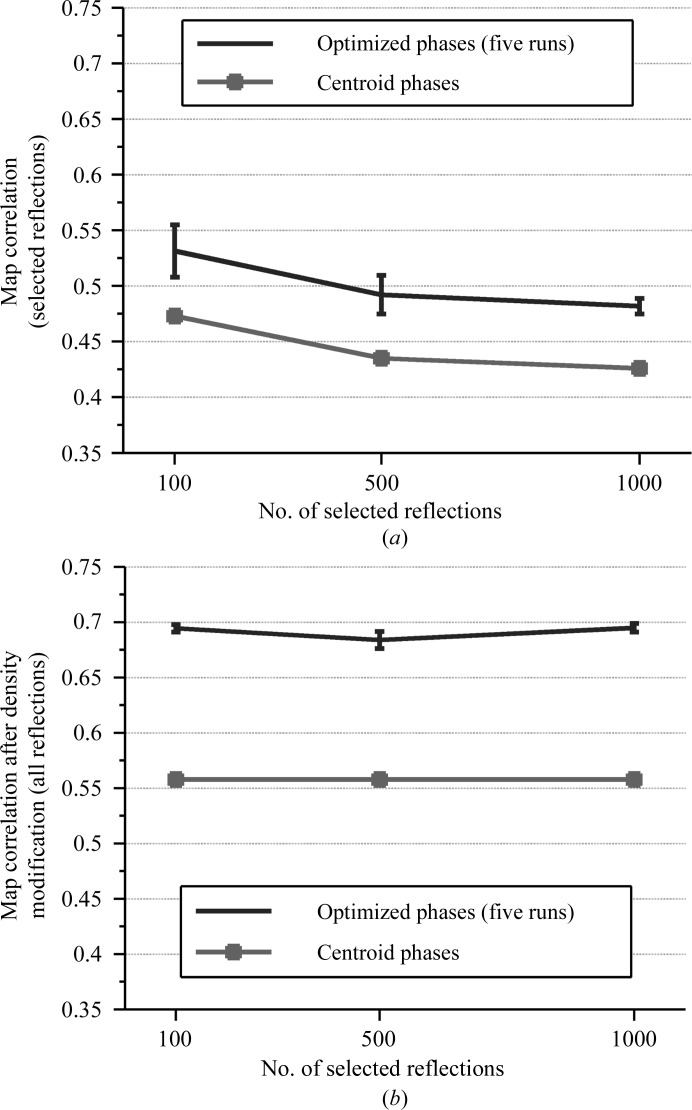
Results of running five independent trials for SAD data from the group II intron to search for phases for the 100, 500 and 1000 selected strongest reflections. (*a*) Map correlations of only the selected reflections for the optimized phases. (*b*) Map correlations of all reflections for the density-modified maps.

The quality of the optimized phases was improved for all of the tests using SAD data for the RNA comprising domains 5 and 6 of the yeast ai5γ group II self-splicing intron (group II intron; case II), regardless of the number of strongest reflections selected (Fig. 9 ▶ *a*). The density-modified maps generated from the new reflection files (with the optimized phases and the remaining centroid phases) were significantly improved; the averaged map correlation for all of the tests (with varying numbers of selected strongest reflections) increased from 0.56 (the map correlation of the density-modified map using the original centroid phases) to 0.70 (Fig. 9 ▶ *b*); this improvement could be obtained with only the 100 strongest reflections optimized.

We noted that subsequent model-building processes resulted in a similar map quality for the data set with only the original centroid phases and for that with both the original centroid phases and the optimized phases. The map correlation of the density-modified maps generated from the centroid phases (0.56) also increased to around 0.7 at the end of these iterative density-modification and model-building cycles: the value that was obtained after one density modification for the data set with the optimized phases included.

**Figure 10:**
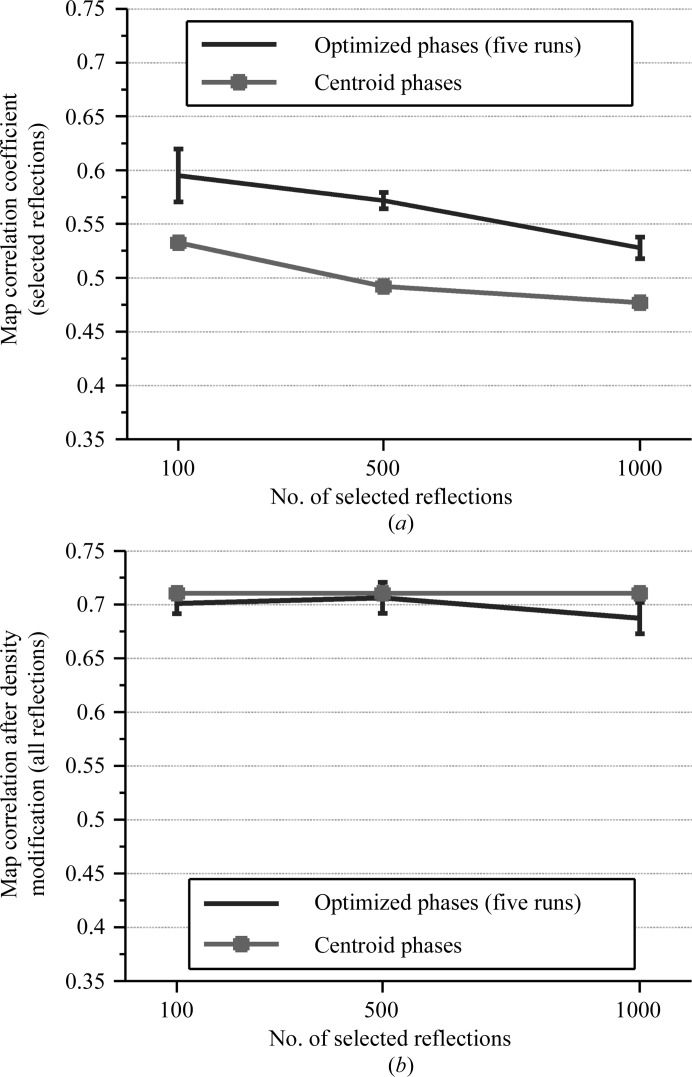
Results of running five independent trials for SAD data for hnRNP. (*a*) Map correlations of only the selected reflections for the optimized phases. (*b*) Map correlations of all reflections for the density-modified maps.

We could obtain phase improvements after the search for the heterogeneous ribonucleoprotein A1 (hnRNP; case III), but no impact was observed on the density-modified maps (Fig. 10 ▶). Similarly to previous test cases, the average improvements of the optimized phases decreased when more reflections were selected. Density modification for all three of the test runs (100, 500 and 1000 strongest reflections) and for the centroid phases yielded similar results with a map correlation of around 0.7. However, an impact on density modification was not observed in this case; this may be because density modification generated from the centroid phases already resulted in a usable map.

### 3.3. Impact of the optimized phases on density modification

**Figure 11:**
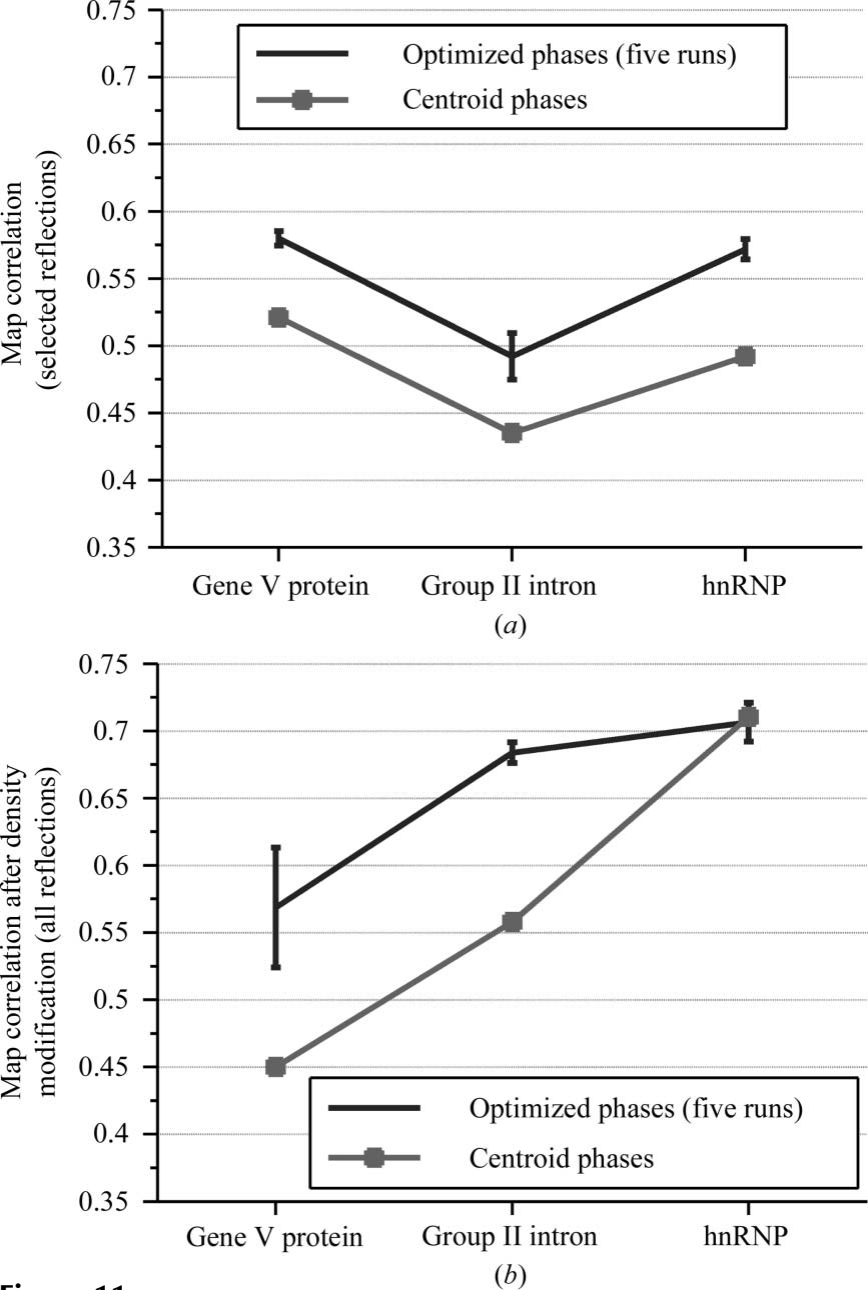
(*a*) Map correlations of the optimized phases and the original centroid phases for the 500 strongest reflections of the three test cases: the gene V protein, the group II intron and hnRNP. (*b*) Map correlations of the density-modified maps generated from the reflections with optimized phases and without optimized phases, respectively.

Depending on the quality of the initial phases, our method can significantly improve density modification and model building. In all three test cases, the map correlations of the strongest reflections were improved after the optimization (the results for the 500 strongest reflections are shown in Fig. 11 ▶ *a*). In the case of hnRNP, however, the density modification had already improved the quality of the original centroid phases to yield a map with reasonably good quality, so that the optimized phases did not provide any further improvement and the search was not necessary. When the density modification had resulted in a less superior map quality, such as in the cases of gene V and group II intron, the optimized phases for the selected strongest reflections had a strong impact on the quality of density modification and the ease of model building (Fig. 11 ▶ *b*).

## 4. Conclusions

Two key ideas have been explored in this work: firstly, reducing the phase errors in a small set of the strongest reflections can have a large impact, and secondly, map skewness is a highly effective measure of phase quality. These ideas were implemented in a computer program, *SISA*, which applies a genetic algorithm to improve the quality of the density map after density modification, leading to greater success in subsequent model building. Results using the three test cases show that the phases of around 1000 selected strongest reflections could be improved through an iterative search using map skewness as the target function. Based on tests that varied the number of strongest reflections (100, 500 or 1000) used in the search, we observed that a greater average phase improvement occurred when smaller numbers of reflections (*e.g.* 100) were selected.

When 100–500 phases were varied and combined with the original centroid phases, ϕ_B_, for the remaining reflections, a large majority of the test runs showed a substantial improvement in the quality of the map after density modification for the group II intron and for the gene V protein. Furthermore, application of the *SISA* procedure greatly facilitated automated model building for the latter.

The calculation time for the search depends on the size of the structures and the numbers of selected reflections. From the three test cases, the smallest structure, the gene V protein, has 682 non-H atoms with around 2500 reflections in space group *C*2. Calculations took about 15 min for the 100 strongest reflections and 1.2 h for the 500 strongest reflections on a 2.4 GHz CPU. The largest structure, group II intron (case II), has 1497 non-H atoms with around 7400 reflections in space group *P*6_1_22. We recorded calculation times of 2 and 10.5 h for 100 and 500 selected reflections, respectively.

Phases optimized by the procedure in *SISA* will be useful for SIR/SAD data sets which produce an electron-density map with marginal quality. *SISA* can be downloaded from http://www.biochem.uni-luebeck.de/public/software/sisa/sisa.html and will be incorporated into the *PHENIX* software suite.

## Figures and Tables

**Table 1:** Summary of data for test proteins

Structure	PDB entry	Space group	Resolution for search (Å)	No. of non-H atoms	Unit-cell parameters (Å, °)
Case I: gene V protein (single-stranded DNA-binding protein; Skinner *et al.*, 1994 ▶)	1vqb	*C*2	2.6	682	*a* = 75.81, *b* = 27.92, *c* = 42.40, β = 103.1
Case II: RNA comprising domains 5 and 6 of the yeast ai5γ group II self-splicing intron (Zhang & Doudna, 2002 ▶)	1kxk	*P*6_1_22	3.5	1497	*a* = *b* = 91.68, *c* = 241.65,
Case III: heterogeneous ribonucleoprotein A1 (Shamoo *et al.*, 1997 ▶)	1ha1	*P*2_1_	3.0	1338	*a* = 38.1, *b* = 44.0, *c* = 56.1, β = 94.8
